# Application of *Trichoderma harzianum* enhances salt tolerance and yield of Indian mustard through increasing antioxidant enzyme activity

**DOI:** 10.1016/j.heliyon.2024.e41114

**Published:** 2024-12-10

**Authors:** Kartik Chandra Saha, Md Kafil Uddin, Pallab Kumer Shaha, Md Akhter Hossain Chowdhury, Lutful Hassan, Biplob Kumar Saha

**Affiliations:** a Department of Agricultural Chemistry, Bangladesh Agricultural University, Mymensingh, 2202, Bangladesh; b Ramdeb Khabir Uddin College, Matinpur, Sundarganj, Gaibandha, 5721, Bangladesh; c Department of Genetics and Plant Breeding, Bangladesh Agricultural University, Mymensingh, 2202, Bangladesh

**Keywords:** Soil salinity, *Trichoderma harzianum*, Antioxidant enzymes, Yield of Indian mustard, Nutrient concentration

## Abstract

Growth and yield reduction of crops due to salt stress have become a serious issue worldwide. *Trichoderma* is very well known as a plant growth-promoting fungi under abiotic stress conditions. Therefore, this study was designed to investigate the effect of *Trichoderma harzianum* on the growth, yield, nutrient uptake, and antioxidant activity of three Indian mustard genotypes under saline condition (EC 9.28 dS m^−1^). A two-factorial (*Trichoderma* and Indian mustard genotypes) pot experiment was conducted following a completely randomized design (CRD) with four replicates. *Trichoderma* was applied to soil as compost and suspension. The BD-7104 genotype showed better performance than Tori-7 under saline conditions. Compared to control, application of *T. harzianum* showed better performance in enhancing growth and yield of all the genotypes by increasing plants' tolerance to salt stress. Again, *Trichoderma* application increased the chlorophyll, proline, and oil content of Indian mustard. The generation of antioxidant enzymes viz., SOD, CAT, APX, and POD was significantly increased and, synthesis of H_2_O_2_ and MDA was decreased to a variable degree under different *Trichoderma* treatments. On average, application of *Trichoderma* as compost enhanced seed yield by 23 % than control. The better growth and yield in *Trichoderma* treated plants were the results of better uptake and assimilation of N, P, S, Ca, Mg, and K and reduced uptake of Na with a lower Na/K. Overall, BD-7104 genotype can be grown in soil treated with *Trichoderma* as compost at a rate of TdC_12.5_ for obtaining better yield and nutritional quality under salinity stress condition.

## Highlights

• *Trichoderma harzianum* enhances salt tolerance of Indian mustard genotypes.
• Application of *Trichoderma* substantially increased antioxidant enzyme activity.
• Soil incorporation of *Trichoderma* enhanced seed yield of Indian mustard by 23 % compared to control.
• *Trichoderma* facilitated better nutrient uptake by Indian mustard.

## Graphical abstract

**Figure:**
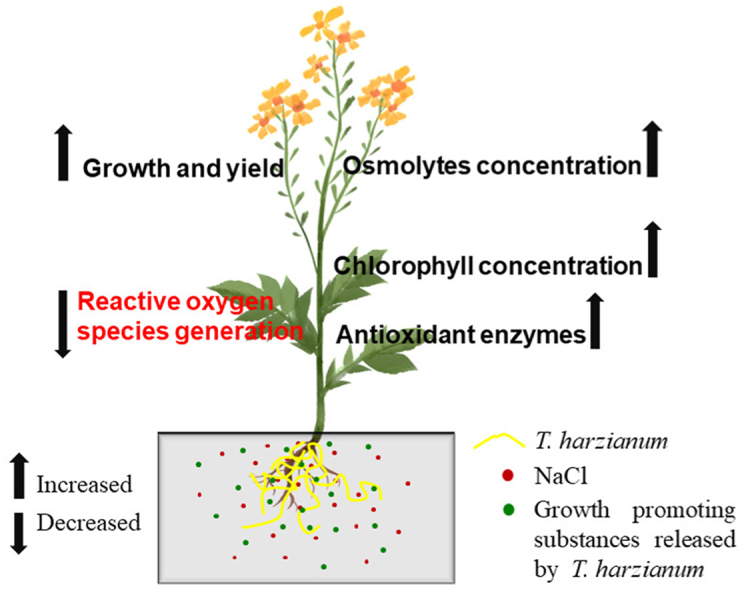

## 1 Introduction

Indian mustard (*Brassica juncea* L.) is a major edible oil crop of rapeseed-mustard (oilseed Brassica) group and is presently being cultivated mainly in the Indian subcontinent and parts of Canada, Russia, China, and Australia [1]. Among the oilseed Brassica group of crops, *B. juncea* is the major oilseed crop of Bangladesh [2]. Besides being used for cooking, Indian mustard has found myriad applications in the food and chemical industry and is also used as a biofertilizer. Mustard seed meal serves as an excellent feed for poultry animals and India has become the largest exporter of mustard seed meal at the global level. Its leaves are consumed as a nutritious vegetable. Mustard oil is an enriched repertoire of various antioxidants and high erucic acid with very good lubrication and combustion properties, hence is quite liked and preferred in biodiesel production in the automobile industry and in the paint industry [3].

Agricultural production worldwide is facing the challenge of meeting the food demand for increasing population size. Food production is increasingly restricted by limited cultivated land due to soil degradation issues, such as salinization. Saline soil accounts for 6 % of the world's total land area [1] and 20 and 30 % of the total cultivated and irrigated area, respectively [4] is a severe hindrance to agricultural production. In Bangladesh, about 1.06 million hectares of cultivated land are salinity-affected, which is about 13.32 % of the total cultivable land. Soil salinization mainly occurred through saline water intrusion due to catastrophic natural hazards like sea levels rising, storm surges, and cyclones in those areas [5]. However, salt accumulation in soil negatively affects the plant physiology and anatomy producing a lot of impacts [6]. Firstly, the root system is affected by the salinity stress causing immediate and long-term changes [7,8]. As an immediate response to salinity, osmotic stress causes the reduction of water availability and latterly hampers plant growth [9]. Consequently, salinity is one of the significant problems among numerous abiotic stresses that have an impact on plant productivity around the world [10]. Again, the increased food demand of current global populations creates a situation to find ways of crop tolerance against salinity [11].

In general, the majority of the crop species are sensitive to salinity. So, the transfer of salinity-tolerant genes to the susceptible crops to induce salt tolerance has been tried like *CODA* gene for encoding choline oxidase enzyme to increase accumulation of betaine by oxidizing choline to betaine, tobacco osmotin (OSM) gene to increase the osmotin induced proline accumulation; but the complex mechanism of salt tolerance and little genetic variation in germplasm resulted in confined or no success in the mitigation of salinity stress [1,12]. Meanwhile, the application of plant growth-promoting microorganisms (PGPM) like endophyte microorganism is also an approach to enhance salt tolerance [[13], [14], [15]]. Among different PGPMs, the avirulent symbiotic endophytic soil fungus *Trichoderma harzianum* was vindicated and highly effective in inducing salinity tolerance in plants [16,17].

During stress, several mechanisms are shown by plants viz., ionic re-equilibrium, reactive oxygen detoxification, balancing cell division, or growth [18]. The colonization of endophytic fungi to crop mitigate salinity stress by increasing the levels of defensive metabolites including osmo-protectants, activation of antioxidant systems for the prevention of damage by reactive oxygen species, and the modulation of phytohormones [19]. They can promote plant growth and improve salt tolerance by increasing effective root area resulting in increased nutrient uptake, solubilization of nutrients, and production of small peptides, volatile compounds, and phytohormones or their analog [[20], [21], [22]].

The genus *Trichoderma* currently contains more than 400 described species that are used in many areas of human life. Among those species, *T. harzianum* is a widely used species for the mitigation of salinity stress [9]. However, the application of *T. harzianum* on wheat plants showed a reduction in the severity of salinity stress [23]. Ahmad et al. [24] reported that the inoculation of *T. harzianum* in Indian mustard showed the restoration of the photosynthetic pigments to a considerable level. Also, the application of *T. harzianum* improved beneficial elements uptake, stimulated the accumulation of compatible solutes, and elevated the antioxidant enzyme levels in *Brassica juncea* L. under salinity stress [24]. Similarly, Rawat et al. [23] reported that *T. harzianum* treated plant showed a lower accumulation of malondialdehyde (MDA), but a higher proline and phenolics content were found under saline conditions. Again, F. Zhang et al. (2019) found that application of *T. harzianum* increased salt tolerance of cucumber (*Cucumis sativus* L) by increasing the activity of antioxidant enzymes viz., peroxidase (POD), polyphenol oxidase (PPO), phenylalanine ammonia-lyase (PAL), catalase (CAT), superoxide dismutase (SOD), ascorbate peroxidase (APX), and glutathione reductase (GR) and increasing the proline, soluble protein, soluble sugar, ascorbic acid, chlorophyll, and improving root activity. They also reported higher growth of cucumber (leaf size, plant height, stem base diameter, root number, root length, fresh weight, and dry weight) by the application of *T. harzianum* under salinity stress.

Therefore, there is strong evidence to apply *Trichoderma* in the mitigation of salinity stress on Indian mustard. To the best of our knowledge, no published work was found on the application of *T. harzianum* as compost and suspension to different salt tolerant Indian mustard genotypes grown in natural saline soil in the context of Bangladesh. So, this experiment was interevent to study the salinity mitigation potential of *T. harzianum* through enhancing salt tolerance, yield, and quality of three contrasting genotypes (Tori-7, BD-7104, and BD-10115) of Indian mustard grown in naturally occurred saline soil.

## 2 Materials and methods

### 2.1 Source of Trichoderma

The *T. harzianum* compost and suspension were collected from Professor Dr. M Bahadur Meah IPM Laboratory, Department of Plant Pathology, Bangladesh Agricultural University, Mymensingh, Bangladesh. *Trichoderma harzianum* was isolated from the root rhizosphere soil of tomato cultivated at the farm site of Bangladesh Agricultural University. The dilution plate technique was used for the isolation of *Trichoderma harzianum*. Subsequently the isolate was identified using the method of Kubicek et al. [70]. Furthermore, the isolate was confirmed as *Trichoderma harzianum* through DNA extraction and gene sequencing technique.

### 2.2 Pot experiment

The experiment was conducted in the pot-house of the Department of Genetics and Plant Breeding, Bangladesh Agricultural University, Mymensingh, Bangladesh during the *Rabi* season (October to March). The elevation from the sea level was 18 m and it faced 24° 43ʹ 20ʺ North latitude and 90° 25ʹ 26ʺ East longitude.

This experiment comprised two factors: one was the Indian mustard genotype and another was *T*. *harzianum* treatment. Three contrasting genotypes of Indian mustard viz., Tori-7, BD-7104, and BD-10115 were used as test crop in this study. The mustard genotypes were selected based on their response to salt stress. Among the 3 studied genotypes of mustard, Tori-7 is susceptible to salinity, BD-10115 is mildly tolerant to salinity and BD-7104 is relatively more tolerant than BD-10115. The seeds of mustard genotypes were collected from the Oilseed Research Centre, Bangladesh Agricultural Research Institute, Bangladesh. Seven *T*. *harzianum* treatment was applied in this experiment viz., Td_0_ = (Without *Trichoderma*; control), TdC_7.5_ = (*Trichoderma* compost; 7.5 g kg^−1^), TdC_10.0_ = (*Trichoderma* compost; 10.0 g kg^−1^), TdC_12.5_ = (*Trichoderma* compost; 12.5 g kg^−1^), TdS_110×10_^5^ = (*Trichoderma* suspension; 110 × 10^5^ cfu), TdS_110×10_^6^ = (*Trichoderma* suspension; 110 × 10^6^ cfu), TdS_110×10_^7^ = (*Trichoderma* suspension; 110 × 10^7^ cfu), respectively. *Trichoderma* compost (mixture of *T*. *harzianum* mycelium with spore and sterilized composted materials) was mixed with soil 7 days before seed sowing and kept in a dark a room. Again, good, healthy, and uniform seeds of three genotypes were dipped in *T*. *harzianum-*suspension for 30 min before sowing. Before *Trichoderma* treatment seeds were treated with 0.5 % sodium hypochlorite for 3 min and washed with normal water. The soil used in this experiment was collected from the saline area of the Batiaghata Upazila in Khulna district (22° 37ʹ 38ʺ North latitude and 89° 31ʹ 36ʺ East longitude). The average electrical conductivity (EC) of the soil was measured by 9.28 dS m^−1^. The soil of this experiment has the following properties: 23.07 % sand; 56.61 % silt; 20.32 % clay; 1.09 % organic carbon; 0.183 % total N; 16.51 μg g^−1^ available P; 0.39 cmol kg^−1^ exchangeable K; 5.23 cmol kg^−1^ exchangeable Ca; 1.42 cmol kg^−1^ exchangeable Mg; 8.57 μg g^−1^ available S; pH = 6.73. About 15 kg of saline soil was used in each pot. The experiment was laid out following a completely randomized design (CRD) with four replications. During pot filling urea, triple superphosphate, muriate of potash, gypsum, boric acid, zinc sulfate, and cow dung were mixed with the soil according to the recommended dose of Fertilizer Recommendation Guide of Bangladesh-2018.

### 2.3 Determination of chlorophyll, proline, and oil content

The chlorophyll *a* and *b* content was measured following the method of Arnon [26]. Representative fresh leaf sample from the top portion of the plant was collected at 45 days after sowing (DAS). The pigment was extracted using 80 % acetone and the absorbance was measured at 645 and 663 nm with a spectrophotometer (T60 U, UK). Leaf proline content was measured following the ninhydrin acid reagent method described by Bates et al. [27]. The solvent extraction method was employed for the estimation of oil content of Indian mustard seeds following the method of Sharma et al. [28] using the Soxhlet apparatus.

### 2.4 Determination of malondialdehyde (MDA) and hydrogen peroxide (H_2_O_2_) content

Lipid peroxidation was determined by measuring the amount of MDA produced by the thiobarbituric acid (TBA) reaction as described in the method of Heath and Packer [29]. Fresh leaf tissue (0.1 g) of Indian mustard was homogenized in a mortar and pestle using fine granular quartz along with 2 mL 0.1 % trichloroacetic acid (TCA) at 4 °C. The homogenate was centrifuged at 12000 rpm for 10 min at 4 °C. Then 1 mL supernatant was taken in a cleaned falcon tube, and 4 mL of reagent mixture (20 % TCA + 0.5 % TBA) was added to that falcon tube and mixed well. The mixture was boiled at 95^o^C for 20 min and quickly cooled in the ice bath to stop the reaction. The mixture was then centrifuged at 5000 rpm for 6 min. The absorbance of the resulting solution was recorded at 532 nm. Hydrogen peroxide was determined from the expanded leaves of the mustard plants. The estimation was done as per the protocol of Velikova et al. [30]. Freshly harvested Indian mustard leaf samples (0.1 g) were homogenized with 3 mL of 0.1 % (w/v) trichloroacetic acid in an ice bath and the homogenate was centrifuged at 12000 rpm for 15 min. Later, 0.5 mL of 50 mM phosphate buffer (pH 7.5) and 1 mL of 1 M potassium iodide (KI) were added to 0.5 mL of the supernatant. The absorbance of the resulting solution was recorded at 390 nm.

### 2.5 Extraction of antioxidant enzymes

Fresh leaves of Indian mustard plants (0.5 g) were homogenized in a mortar and pestle using fine granular quartz for making grinding easy and efficient with 10 mL phosphate buffer (PB 50 mM, pH 7.5) under chilled conditions according to the procedure of Ediga et al. [31]. The homogenate was filtered through muslin cloth and centrifuged at 12000 rpm for 10 min. After centrifugation, 5 mL of clean and clear supernatant was used for the determination of each of the following enzyme.

### 2.6 Assays of antioxidant enzymes

#### 2.6.1 Superoxide dismutase (SOD)

Superoxide dismutase activity was determined as described by Beyer Jr and Fridovich [32] through measuring its ability to inhibit the photochemical reduction of nitroblue tetrazolium chloride (NBT). The absorbance of the reaction mixture was recorded at 560 nm (UV-1800, Shimadzu, Japan) and the results were expressed as μmol g^−1^ FW.

#### 2.6.2 Catalase (CAT)

Catalase (CAT) was measured following the method of Aebi [33]. The assay mixture contained phosphate buffer (PB 50 mM, pH 7.5), EDTA (2.5 mM), H_2_O_2_ (0.6 %), and enzyme extract in the cuvette, and the absorbance was measured at 240 nm (UV-1800, Shimadzu, Japan). Catalase content was expressed as μmol min^−1^g^−1^ fresh weight. A cuvette containing all reagents except enzyme extract was used as a reagent blank.

#### 2.6.3 Ascorbate peroxidase (APX)

Ascorbate peroxidase (APX) was determined according to the method of Nakano and Asada [34]. The reaction mixture (1 mL) contained phosphate buffer (50 mM, pH 7.5), EDTA (2.5 mM), ascorbate (2.5 mM), H_2_O_2_ (0.6 %), and enzyme extract. The reaction was started by the addition of H_2_O_2_ and ascorbate oxidation was measured at 290 nm (UV-1800, Shimadzu, Japan) for 1 min. The same volume of all reagents contained in a cuvette except enzyme extract was used as a reagent blank.

#### 2.6.4 Peroxidase (POD)

Peroxidase (POD) was measured following the method of Kar and Mishra [35]. The assay mixture contained (1.0 mL) phosphate buffer (PB 50 mM, pH 7.5), EDTA (2.5 mM), H_2_O_2_ (0.6 %), guaiacol (0.1 %) and enzyme extract in the cuvette. The absorbance was measured at 470 nm (UV-1800, Shimadzu, Japan). A cuvette containing all reagents of the same volume except enzyme extract was used as a reagent blank.

### 2.7 Sample processing and nutrient determination

Oven dried and finely ground plant samples were used for the determination of N, P, S, Ca, Mg, K, and Na. The N concentration was determined by adopting the semi-micro-Kjeldhal method described by Bremner and Mulvaney [36]. Phosphorus was determined spectrophotometrically (T60U, UK) following Page et al. [37]. Calcium and Mg were determined by the complexometric method of titration described by Page et al. [37]. Potassium and Na were measured using a flame photometer (JENWAY-PFP7, UK) following the method of Knudsen et al. [38].

### 2.8 Plant growth and yield parameters

Plant height, branches, and siliqua per plant were recorded at the physiological maturity stage (maximum dry matter accumulation period; determined by visual basis by observing siliqua and fixed at 60 DAS). Tori-7 was harvested at 78 DAS and BD-7104, and BD-10115 were harvested at 89 DAS. The seed and stover yield were measured after harvesting.

### 2.9 Statistical analysis

The data of this experiment was analyzed using the statistical software Minitab 18®. A two-way ANOVA was performed to analyze the data considering the factor Indian mustard genotype and *Trichoderma* treatment. Before performing the ANOVA, Kolmogorov-Smirnov and modified Levene's test was done to confirm whether the data were normally distributed or not and also to check equal variance. The significant differences and comparisons among different treatments were performed using Tukey's HSD test at 5 % level of significance.

## 3 Results

### 3.1 Chlorophyll, proline, and oil content

**Fig. 1:**
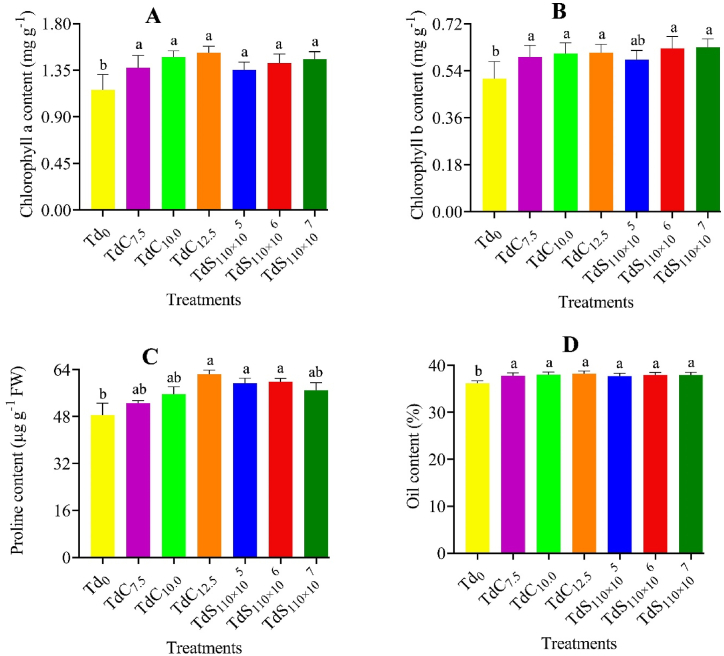
Chlorophyll *a* (A), chlorophyll *b* (B), proline (C), and oil (D) content under different *T. harzianum* treatments. The bars are made using mean ± standard error of the mean. The same letter on the treatment bar indicates the statistical similarity among them as per Tukey's test at p < 0.05.

A significant (p < 0.05) variation was observed among the genotype and *Trichoderma* level on chlorophyll, proline and oil content of Indian mustard (SI Fig. 1). A significantly higher chlorophyll (Fig. 1 A and B), proline (Fig. 1 C) and oil (Fig. 1 D) content was found in the BD-7104 genotype than Tori-7 genotype. The application of *Trichoderma* compost and suspension significantly increased the chlorophyll, proline and oil content of Indian mustard over control. A significantly higher chlorophyll, proline and oil content was found in the TdC_12.5_ treatment that was identical to all the *Trichoderma* treatments except control. Although the interactions of Indian mustard genotype and *Trichoderma* level didn't show any significant effect on chlorophyll (Chl a and Chl b), proline, and oil content (Table 1). The highest values of chlorophyll, proline and oil content was obtained from the BD-7104 genotype treated with *Trichoderma* compost at a rate of 12.5 g per kg soil.

**Table 1:** Interaction effects of different *T. harzianum* treatment and Indian mustard genotypes on the chlorophyll *a*, chlorophyll *b*, proline, and oil content of Indian mustard.

Genotype	*Trichoderma* levels	Chlorophyll (mg g^−1^)		Proline content (μg g^−1^ FW)	Oil content (%)
		Ch a	Ch b		
Tori-7	Td_0_	1.11	0.56	47.44	34.93
	TdC_7.5_	1.17	0.61	51.81	36.08
	TdC_10.0_	1.28	0.61	53.29	36.17
	TdC_12.5_	1.31	0.67	53.82	36.42
	TdS_110×10_^5^	1.21	0.59	50.97	35.91
	TdS_110×10_^6^	1.26	0.69	52.99	36.27
	TdS_110×10_^7^	1.27	0.68	52.98	36.26
BD-7104	Td_0_	1.37	0.59	53.17	37.89
	TdC_7.5_	1.58	0.66	60.75	39.73
	TdC_10.0_	1.59	0.67	61.92	39.99
	TdC_12.5_	1.62	0.69	65.06	40.11
	TdS_110×10_^5^	1.47	0.64	60.43	39.67
	TdS_110×10_^6^	1.56	0.65	62.64	39.78
	TdS_110×10_^7^	1.57	0.65	62.63	39.79
BD-10115	Td_0_	1.24	0.48	51.88	36.47
	TdC_7.5_	1.33	0.51	58.26	37.73
	TdC_10.0_	1.37	0.52	59.37	37.93
	TdC_12.5_	1.41	0.55	62.07	38.25
	TdS_110×10_^5^	1.35	0.52	56.98	37.66
	TdS_110×10_^6^	1.37	0.54	60.04	37.92
	TdS_110×10_^7^	1.38	0.54	60.03	37.92
Significance		NS	NS	NS	NS

Td = *Trichoderma*, TdC = *Trichoderma* compost, TdS = *Trichoderma* suspension.

NS indicates non-significant at 5 % level of significance.

### 3.2 Antioxidant and toxic compound

**Fig. 2:**
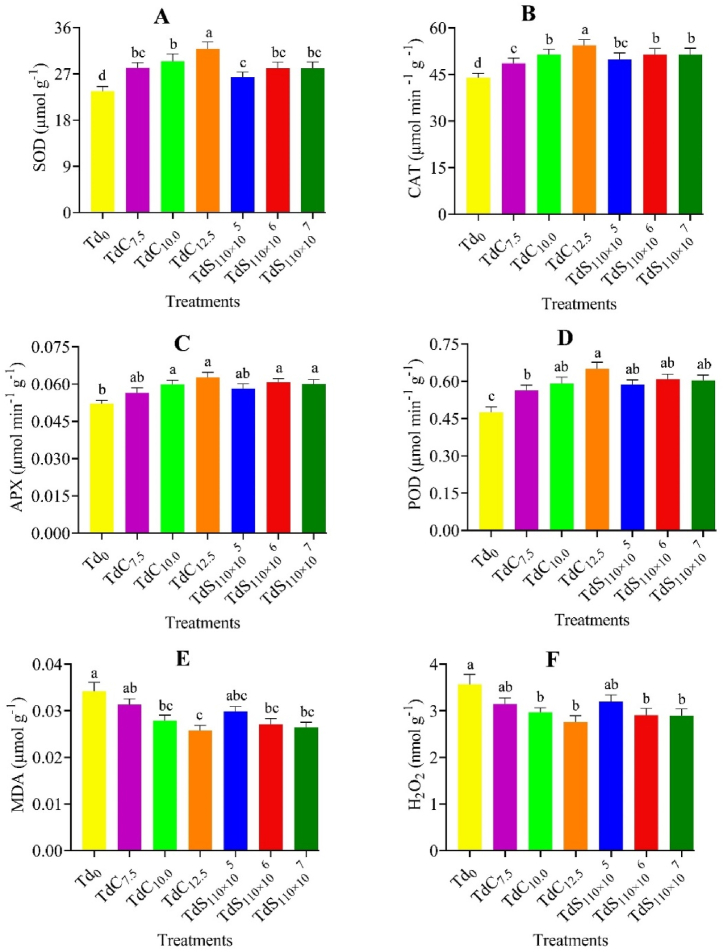
SOD = Superoxide dismutase (A), CAT = Catalase (B), APX = Ascorbate peroxidase (C), POD = Peroxidase (D), MDA = Malondialdehyde (E), and H_2_O_2_ (F) content under different *T. harzianum* treatment. The bars are made using mean ± standard error of the mean. The same letter on the treatment bar indicates the statistical similarity among them as per Tukey's test at p < 0.05.

The superoxide dismutase (SOD), catalase (CAT), and peroxidase (POD) significantly (p < 0.05) varied among the genotypes (SI Fig. 2). The SOD content was significantly higher in Tori-7 genotype than BD-7104 genotypes. On the other hand, the CAT, APX and POD content were significantly higher in BD-7104 genotype compared to Tori-7 genotype. However, the toxic compound production was also significantly (p < 0.05) varied among the three genotypes and showed a different trend in relation to antioxidant enzymes. Tori-7 genotype produced the highest malondialdehyde (MDA) and hydrogen peroxide (H_2_O_2_) compared to BD-7104 genotype. The application of *Trichoderma* as compost and suspension under saline conditions showed a significant (p < 0.05) effect on antioxidant enzymes and toxic compounds production by Indian mustard genotypes. The highest SOD (Fig. 2 A) and CAT (Fig. 2 B) production was measured from the application of TdC_12.5_ which was significantly different from all other treatments except TdC_10_ treatment. As expected, the control treatment showed the lowest values of SOD and CAT. However, the APX production didn't significantly vary among the *Trichoderma* treatments except the control treatment which showed a significantly lower APX production (Fig. 2 C). The highest POD production was measured from the application of TdC_12.5_ and the lowest POD was obtained from the control (Fig. 2 D). Again, the application *Trichoderma* showed a significant (p < 0.05) reduction of toxic compound production in Indian mustard. The lowest MDA production was found in TdC_12.5_ (0.026 μmol g ^−1^) which was identical to TdC_10.0_, TdS_110__× 10_^5^, TdS_110__× 10_^6^, and TdS_110__× 10_^7^, respectively (Fig. 2 E). The highest MDA (0.034 μmol g ^−1^) was found in the control treatment. The H_2_O_2_ production didn't vary among different *Trichoderma* treatments (Fig. 2 F). The interactions of genotype and *Trichoderma* level failed to show any significant effect on antioxidants and toxic compounds production of Indian mustard genotypes (Table 2). However, the highest values of CAT, APX and POD were obtained from the BD-7104 genotype receiving Trichoderma as compost (TDC_12.5_). On the other hand, the treatment combination of Tori-7 and TDC_12.5_ showed the best production SOD and H_2_O_2_.

**Table 2:** Interaction effects of different *T. harzianum* treatment and Indian mustard genotypes on the antioxidant enzymes concentration and toxic compounds of Indian mustard.

Genotype	*Trichoderma* levels	Antioxidant enzymes				Toxic compounds	
		SOD (μmol g^−1^)	CAT (μmol min^−1^ g^−1^)	APX (μmol min^−1^ g^−1^)	POD (μmol min^−1^ g^−1^)	MDA (μmol g^−1^)	H_2_O_2_ (nmol g^−1^)
Tori-7	Td_0_	26.33	40.73	0.052	0.46	0.037	3.73
	TdC_7.5_	31.65	44.97	0.057	0.55	0.034	3.31
	TdC_10.0_	34.74	47.97	0.059	0.58	0.030	3.11
	TdC_12.5_	36.86	49.76	0.061	0.65	0.027	2.98
	TdS_110×10_^5^	29.81	44.89	0.058	0.59	0.031	3.39
	TdS_110×10_^6^	32.19	46.99	0.060	0.61	0.028	3.18
	TdS_110×10_^7^	32.18	46.98	0.060	0.61	0.028	3.17
BD-7104	Td_0_	21.47	49.35	0.053	0.53	0.031	3.42
	TdC_7.5_	25.24	55.04	0.057	0.62	0.029	2.98
	TdC_10.0_	25.56	57.74	0.062	0.66	0.026	2.83
	TdC_12.5_	28.17	61.52	0.066	0.72	0.024	2.55
	TdS_110×10_^5^	23.68	58.23	0.059	0.63	0.028	3.01
	TdS_110×10_^6^	24.71	58.67	0.062	0.65	0.025	2.65
	TdS_110×10_^7^	24.70	58.67	0.062	0.65	0.025	2.64
BD–10115	Td_0_	23.19	41.82	0.051	0.43	0.034	3.57
	TdC_7.5_	27.61	45.86	0.055	0.51	0.031	3.14
	TdC_10.0_	28.08	48.61	0.058	0.53	0.027	2.96
	TdC_12.5_	30.63	51.77	0.060	0.57	0.026	2.75
	TdS_110×10_^5^	25.63	46.31	0.057	0.54	0.030	3.19
	TdS_110×10_^6^	27.46	48.74	0.059	0.56	0.028	2.89
	TdS_110×10_^7^	27.43	48.71	0.059	0.56	0.027	2.88
Significance		NS	NS	NS	NS	NS	NS

Td = *Trichoderma*, TdC = *Trichoderma* compost, TdS = *Trichoderma* suspension, SOD = Superoxide dismutase, CAT = Catalase, APX = Ascorbate peroxidase, POD = Peroxidase, MDA = Malondialdehyde, H_2_O_2_ = Hydrogen peroxide.

NS indicates non-significant at 5 % level of significance.

### 3.3 Growth, yield attributes, and yield

A significant (p < 0.05) variation was found among the genotypes in case of growth, yield attributes, and yield (SI Table 1). Plant height, number of branches, number of siliqua, seed and stover yield were significantly higher in BD-7104 genotype compared to Tori-7 genotype. Again, the application of *Trichoderma* compost and suspension significantly (p < 0.05) increased growth, yield attributes, and yield of Indian mustard compared to the control soil not receiving any form of *Trichoderma* (SI Table 1). The highest plant height, number of branches, number of siliqua, seed and stover yield were found in TdC_12.5_ treatment and as obvious the lowest values were measured from the control treatment.

No significant interaction was noticed between genotypes and *Trichoderma* treatments on the growth, yield attributes, and yield of Indian mustard (Table 3). However, like other parameters the treatment combination of BD-7104 genotype and application *Trichoderma* compost at a rate of 12.5 g kg^−1^ soil showed better performance compared to the other genotype and *Trichoderma* treatment combinations.

**Table 3:** Interaction effects of different *T. harzianum* treatment and Indian mustard genotypes on the growth, yield contributing characters, and yield of Indian mustard.

Genotype	*Trichoderma* levels	Plant height (cm)	No. of branches plant ^−1^	No. of siliqua plant ^−1^	Seed yield pot ^−1^	Stover yield pot ^−1^
Tori-7	Td_0_	43.08	4.00	113	4.53	9.27
	TdC_7.5_	50.14	4.33	136	5.51	10.26
	TdC_10.0_	50.37	5.00	159	5.86	11.25
	TdC_12.5_	50.95	5.33	178	5.89	11.48
	TdS_110×10_^5^	44.23	4.33	143	5.36	10.38
	TdS_110×10_^6^	46.43	5.00	170	5.64	11.47
	TdS_110×10_^7^	44.98	4.67	175	5.75	11.05
BD-7104	Td_0_	62.91	4.33	218	9.24	17.01
	TdC_7.5_	64.22	5.33	243	10.02	21.20
	TdC_10.0_	65.15	5.33	254	10.73	21.26
	TdC_12.5_	65.71	5.67	256	10.99	24.21
	TdS_110×10_^5^	63.34	5.00	230	9.97	19.74
	TdS_110×10_^6^	63.81	5.67	244	10.42	22.51
	TdS_110×10_^7^	63.76	5.33	234	10.16	20.79
BD-10115	Td_0_	59.14	4.33	206	7.64	14.15
	TdC_7.5_	62.19	4.33	237	8.56	16.50
	TdC_10.0_	64.11	5.00	247	8.9	18.57
	TdC_12.5_	64.42	5.33	249	9.16	19.30
	TdS_110×10_^5^	60.37	4.67	225	8.17	15.92
	TdS_110×10_^6^	62.47	5.33	236	8.62	18.83
	TdS_110×10_^7^	61.31	5.00	231	8.45	16.58
Significance		NS	NS	NS	NS	NS

Td = *Trichoderma*, TdC = *Trichoderma* compost, TdS = *Trichoderma* suspension.

NS indicates non-significant at 5 % level of significance.

### 3.4 Nutrient concentration

The nutrient concentration was significantly (p < 0.05) influenced by the individual effect of genotypes and different *Trichoderma* treatments (SI Fig. 3). The N, P, K, Ca and Mg concentrations were significantly higher in BD-7104 genotype than Tori-7 genotype whereas S concentration didn't vary among genotypes. In contrast, a significantly higher Na concentration and Na/K ratio were found in Tori-7 genotype than BD-7104 genotype.

**Fig. 3:**
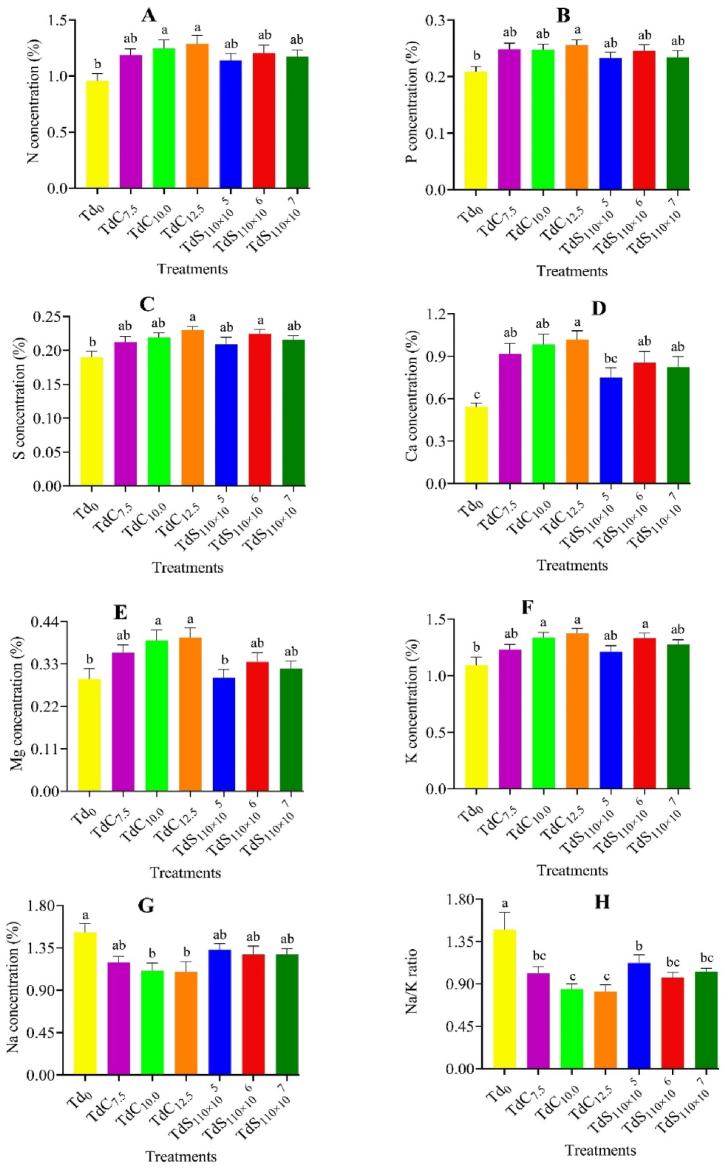
Biomass N (A), P (B), S (C), Ca (D), Mg (E), K (F), Na (G) concentration and Na/K (H) ratio under different *T. harzianum* treatments. The bars are made using mean ± standard error of the mean. The same letter on the treatment bar indicates the statistical similarity among them as per Tukey's test at p < 0.05.

The application of *Trichoderma* compost and suspension exerted a significant (p < 0.05) effect on the mineral nutrient concentration of Indian mustard under saline conditions. The highest N, P, K, Ca, Mg and S concentrations were determined from the plants receiving *Trichoderma* compost at a rate of 12.5 g per kg soil and the lowest values were obtained from the control treatment (Fig. 3 A–F). However, Na concentration and Na/K ratio were significantly (p < 0.05) decreased in different *Trichoderma* treatments than control (Fig. 3 G and H). A significantly lower Na concentration and Na/K ratio was found in TdC_12.5_ treatment than control which was identical to all other *Trichoderma* treatments. Again, the interaction of genotypes and different *Trichoderma* treatments showed a significant variation on the Na/K ratio whereas the interaction effect was not significant for the measured mineral nutrients (Table 4). A significantly higher Na/K ratio was found in Tori-7 genotype grown in control soil. The lowest Na/K ratio was found the treatment combination of BD-7104 and TdC_12.5_. On the other hand, the highest concentrations of N, P, K, Ca, Mg and S were determined from the BD-7104 genotype receiving *Trichoderma* compost at a rate of 12.5 g per kg soil.

**Table 4:** Interaction effects of different *T. harzianum* treatment and Indian mustard genotypes on the growth, yield contributing characters, and yield of Indian mustard. The vales express mean ± standard error of mean (n = 3). The same letter on the treatments values indicates the statistically similarity among them as per Tuky's test at p < 0.05.

Genotype	*Trichoderma* levels	Nutrient concentration (%)							Na/K
		N	P	S	Ca	Mg	K	Na	
Tori-7	Td_0_	0.81	0.18	0.17	0.56	0.22	0.88	1.86	2.14 ± 0.14 a
	TdC_7.5_	1.18	0.23	0.20	0.90	0.31	1.15	1.40	1.23 ± 0.12 bc
	TdC_10.0_	1.24	0.24	0.22	0.96	0.34	1.30	1.20	0.92 ± 0.04 bc
	TdC_12.5_	1.24	0.25	0.23	0.97	0.34	1.34	1.15	0.85 ± 0.11 bc
	TdS_110×10_^5^	1.07	0.22	0.18	0.71	0.25	1.13	1.45	1.31 ± 0.19 b
	TdS_110×10_^6^	1.14	0.23	0.22	0.79	0.28	1.32	1.28	0.96 ± 0.06 bc
	TdS_110×10_^7^	1.11	0.21	0.22	0.77	0.27	1.25	1.32	1.06 ± 0.03 bc
BD-7104	Td_0_	1.11	0.22	0.21	0.60	0.37	1.31	1.30	0.99 ± 0.02 bc
	TdC_7.5_	1.28	0.27	0.23	1.09	0.39	1.34	1.13	0.79 ± 0.01 bc
	TdC_10.0_	1.34	0.26	0.23	1.15	0.42	1.41	1.10	0.77 ± 0.10 c
	TdC_12.5_	1.45	0.26	0.22	1.15	0.42	1.43	1.06	0.78 ± 0.10 bc
	TdS_110×10_^5^	1.32	0.25	0.23	0.94	0.33	1.33	1.22	0.91 ± 0.07 bc
	TdS_110×10_^6^	1.38	0.26	0.22	1.06	0.39	1.37	1.19	0.88 ± 0.09 bc
	TdS_110×10_^7^	1.35	0.27	0.22	1.03	0.36	1.36	1.22	0.92 ± 0.04 bc
BD–10115	Td_0_	0.96	0.22	0.19	0.47	0.28	1.10	1.38	1.26 ± 0.01 bc
	TdC_7.5_	1.10	0.25	0.21	0.76	0.37	1.20	1.06	0.88 ± 0.06 bc
	TdC_10.0_	1.16	0.24	0.21	0.84	0.42	1.30	1.02	0.80 ± 0.18 bc
	TdC_12.5_	1.18	0.25	0.23	0.93	0.43	1.35	1.08	0.78 ± 0.11 c
	TdS_110×10_^5^	1.03	0.23	0.22	0.60	0.30	1.18	1.32	1.12 ± 0.04 bc
	TdS_110×10_^6^	1.08	0.24	0.23	0.71	0.33	1.30	1.36	1.04 ± 0.14 bc
	TdS_110×10_^7^	1.06	0.23	0.21	0.68	0.32	1.22	1.30	1.06 ± 0.09 bc
Significance		NS	NS	NS	NS	NS	NS	NS	∗∗

Td = *Trichoderma*, TdC = *Trichoderma* compost, TdS = *Trichoderma* suspension.

NS indicates non-significant at 5 % level of significant and ∗∗ indicates significant at 5 % and 1 % level of significance.

## 4 Discussion

*Trichoderma* is a soil-borne fungus that colonizes plant roots and directly or indirectly accords interactions among soil, plant, and environment [39]. This experiment investigates the salt stress mitigation potential of *T. harzianum* on Indian mustard genotypes grown under saline conditions. In this experiment, Tori-7 genotype showed relatively lower performance than BD-7104 and BD-10115 genotypes concerning all studied parameters. However, *T. harzianum* application ameliorated the salinity stress to Tori-7 genotype and showed a greater improvement than the control treatment. The chlorophyll and oil content of Tori-7 was substantially lower compared to the other two genotypes (SI Fig. 1A–D). This might be due to the susceptibility of Tori-7 to salinity than the other two genotypes. Interestingly no significant variation in proline content was observed among the three genotypes. Again, the application of *T. harzianum* as compost and suspension showed an increase in chlorophyll, proline, and oil content than the control treatment (Fig. 1 A–D). Under salt stress condition plant accumulates osmolytes, soluble sugar [40] and proline [41] to adjust the osmotic balance that prevents the plant from dehydration under physiological drought. Proline helps to degrade reactive oxygen species (ROS), stabilizes cell membranes, and prevents the protein and enzymes from breaking down under salt stress [42]. In a previous experiment, Zhang et al. [25] reported that *T. harzianum* application increases the proline content of cucumber (*Cucumis sativus* L) seedlings under salt stress. Several published literature showed that salt stress reduces the chlorophyll content due to inhibition of synthesis from enzyme breakdown and degeneration of pigment-protein complexes [43,44] and reducing the supply of nutrients (Mg^2+^, Mn^2+^, and Zn^2+^) [45,46]. The increase of chlorophyll content in our experiment may be a result of higher uptake of nutrients especially Mg^2+^ and synthesis of phytohormones rewarded from *Trichoderma* application. Abdelrhim et al. [47] showed that *T. koningii* and *T. harzianum* application mitigated saline stress in common bean (*Phaseolus vulgaris*) and increased the chlorophyll *a* and *b* content. The oil content significantly increased in all *Trichoderma* treatments might be due to the reduction of abscisic acid production under salt stress which enables cytokinin to transport from root to shoot of plant [48,49] because cytokinin is important for pod formation, seed development, and seed size of oilseed crop [50]. In line with our findings, Ahmad et al. [24] found that *T. harzianum* application in Indian mustard increased oil content by 19–23.4 % under NaCl stress.

The varietal/cultivar variation in the generation of reactive species (ROS, MDA and H_2_O_2_) has been addressed already and the results were in line with the abovementioned results (SI Fig. 2A–F). However, under *Trichoderma* treatment, the ROS production was higher in the control treatment, and the production rate was decreased significantly and other variable degrees in all other treatments. Our results correlate with the findings of Metwally and Soliman [51] who found that the generation of MDA and H_2_O_2_ decreased by around 14 and 24.8 % due to *T. viride* application on tomato seedlings (*Solanum Lycopersicum* L.) under salt stress. Salinity stress induces the plant to produce ROS and MDA [52,53] which can modify the components of cells and molecules; for example, they cause oxidation of carbohydrates, proteins, lipids, DNA, and enzymes and result in programmed cell death [54,55]. Plants use several antioxidant enzymes to regulate the production of ROS to prevent damage is achieved by either directly detoxifying the ROS or by restoring the number of endogenous antioxidants [56,57]. It is well reported that *Trichoderma* can increase the activity and content of antioxidant enzymes under salt stress [[58], [59], [60]]. The higher concentration of SOD, CAT, APX, and POD in *Trichoderma* treated plants in this experiment reflects the beneficial association of *Trichoderma* with Indian mustard genotypes under salt stress (Fig. 2 A–F). This beneficial effect is also obvious from the increase of antioxidant enzymes of Tori-7 in comparison to the other genotypes. In a previous experiment, Abdelrhim et al. [47] showed that SOD, CAT, POD, and APX of common bean (*Phaseolus vulgaris*) increased under saline conditions due to the application of *T. koningii* and *T. harzianum*.

Soil salinity affects plant growth and yield due to water unavailability, poor root growth resulting in poor shoot growth, and oxidative damage of cells and tissues [61]. In this experiment, the growth and yield reduction were higher in Tori-7 genotype than the other two genotypes. However, the application of *Trichoderma* substantially reduced the salt stress and enhanced growth and yield of Indian mustard grown under saline conditions. The higher yield increase (25.13 %) was noticed in Tori-7 genotype compared to other genotypes. Interestingly, *Trichoderma* application showed a considerable effect on the yield improvement of the Indian mustard genotypes under saline conditions. The beneficial effect of *Trichoderma* in this experiment presumably resulted from higher proline and antioxidant enzyme accumulation that increases the osmotic potential and reduces the activity of ROS and toxic compounds in the plant; which reduces the cell and tissue damage and maintains proper root and shoot growth of plant. The higher chlorophyll content is also another indicator of stress reduction and vigorous metabolisms in plants. Here also another finding is that *Trichoderma* compost showed better performance than suspension. Compost is well known for the increase of crop growth and yield due to the improvement of soil physical, chemical, and biological properties that increase soils’ capacity to reduce Na^+^ toxicity to plants by exchanging Na^+^ with other cations like K^+^, Ca^2+^, and Mg^2+^ that cause leaching Na^+^ from saline soil [62,63]. The beneficial effect of *Trichoderma* spp. was stated by Kumar et al. [64] on maize (*Zea mays* L.) where they found that root and shoot length, leaf area, total biomass, stem, and leaf fresh weight significantly increased under saline conditions. Again, Singh et al. [65] reported a significant increase in plant height, number of tillers per plant, and biomass yield under saline-sodic soil conditions from the combined application of *T. harzianum* and *Bacillus amyloliquefaciens*.

Again, the variation in nutrient concentrations of Indian mustard genotypes showed similar trend like growth and yield parameters. However, *Trichoderma* application showed a considerable increase in nutrient (N, P, S, Ca, Mg, and K) concentrations of Indian mustard genotypes (Fig. 3 A–F). The overall stress reduction and better plant growth might be responsible for the higher uptake of nutrients under saline conditions. Our findings were confirmed by Sofy et al. [66], who found that *Trichoderma* application increased the N, P, and K concentrations of spinach (*Spinacia oleracea* L.) under saline conditions. Again, Na concentration and Na/K ratio were decreased at a variable rate under *Trichoderma* application than the control treatment (Fig. 3 G and H). The decrease in the Na/K ratio is mainly due to the increase in K uptake by plants. Here also interesting that, BD-7104 genotypes showed lower tissue Na concentration and Na/K ratio and higher K concentration than Tori-7 genotypes might be due to its genetic tolerance capacity to salinity. Genetically tolerant plants benefited more from ion homeostasis due to Na^+^ exclusion by transporter and more K^+^ loading to the xylem vessels [67]. Khamesi et al. [68] reported higher antioxidant activity and ion homeostasis ability as a reason for salt tolerance of chickpea genotype ILC-482. Again, the issue of better performance of *Trichoderma* compost than suspension has already been addressed. Our results correlate with the findings of Abdelrhim et al. [47], Sofy et al. [66], and S. Zhang et al. [69], where they found that *Trichoderma* application under salt stress condition increased K uptake and decreased Na uptake and Na/K ratio in the plant.

## 5 Conclusion

Successful crop production by mitigating the rising salinity problems of soils is becoming a challenge due to ongoing climate change. As an eco-friendly approach, *Trichoderma* application has become an appreciable place nowadays. In this experiment, application of *T. harzianum* as compost at a rate of 12.5 g kg^−1^ soil showed the best salt tolerance capacity of BD-7104 genotype of Indian mustard through increasing the osmolytes (proline) and antioxidant enzymes activity (SOD, CAT, APX, and POD), which helps to reduce the toxic H_2_O_2_ and MDA generation. As a result, growth, yield, and nutrient concentration of BD-7104 genotype increased and decreased the Na concentration and Na/K ratio in plants. Therefore, cultivation of BD-7104 genotype in moderately saline soil amended with *T. harzianum* as compost at a rate of 12.5 g kg^−1^ soil could be a best strategy to enhance salinity tolerance for obtaining higher yield and nutritional quality of Indian mustard. So, it can be considered as a baseline data to apply *T. harzianum* in the coastal regions of Bangladesh to mitigate the salinity stress for successful cultivation of Indian mustard. Of course, more experiments need to be conducted under field conditions before making any final recommendation.

## CRediT authorship contribution statement

**Kartik Chandra Saha:** Writing – original draft, Project administration, Methodology, Formal analysis, Data curation, Conceptualization. **Md Kafil Uddin:** Resources, Project administration, Methodology, Investigation, Formal analysis, Conceptualization. **Pallab Kumer Shaha:** Writing – original draft, Visualization, Software, Methodology, Investigation, Formal analysis, Data curation. **Md Akhter Hossain Chowdhury:** Writing – review & editing, Supervision, Software, Resources, Project administration, Funding acquisition, Data curation, Conceptualization. **Lutful Hassan:** Writing – review & editing, Validation, Software, Resources, Project administration, Investigation, Data curation. **Biplob Kumar Saha:** Writing – review & editing, Validation, Supervision, Resources, Project administration, Funding acquisition.

## Data availability

Data will be made available on request.

## Declaration of competing interest

The authors declare that they have no known competing financial interests or personal relationships that could have appeared to influence the work reported in this paper.
