# Supplementary material for: Application of Trichoderma harzianum enhances salt tolerance and yield of Indian mustard through increasing antioxidant enzyme activity
Source: Heliyon. 2024 Dec 10;11(1):e41114. doi: 10.1016/j.heliyon.2024.e41114 (PMC11699397; doi:10.1016/j.heliyon.2024.e41114)
Supplement: Multimedia component 1 [file mmc1.docx]

**Application of *Trichoderma harzianum* enhances salt tolerance and yield of Indian mustard through increasing antioxidant enzyme activity**

**Supporting Information**

Total pages: 5

Number of Tables: 1

Number of Figures: 3


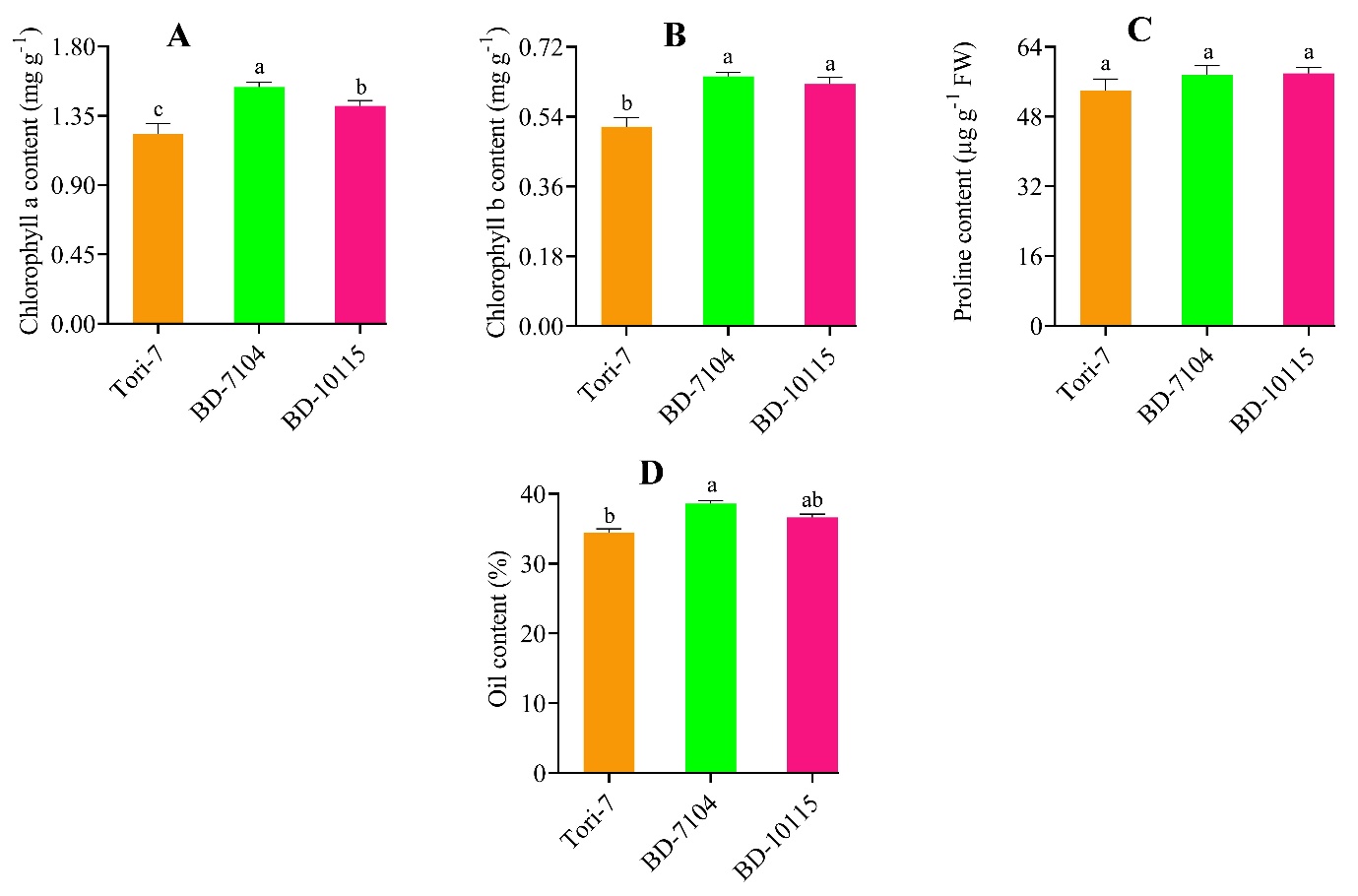


**SI Fig. 1.** Chlorophyll a, chlorophyll b, proline, and oil content of Indian mustard genotypes. The bars are made using mean ± standard error of the mean. The same letter on the treatment bar indicates the statistical similarity among them as per Tukey’s test at p < 0.05.


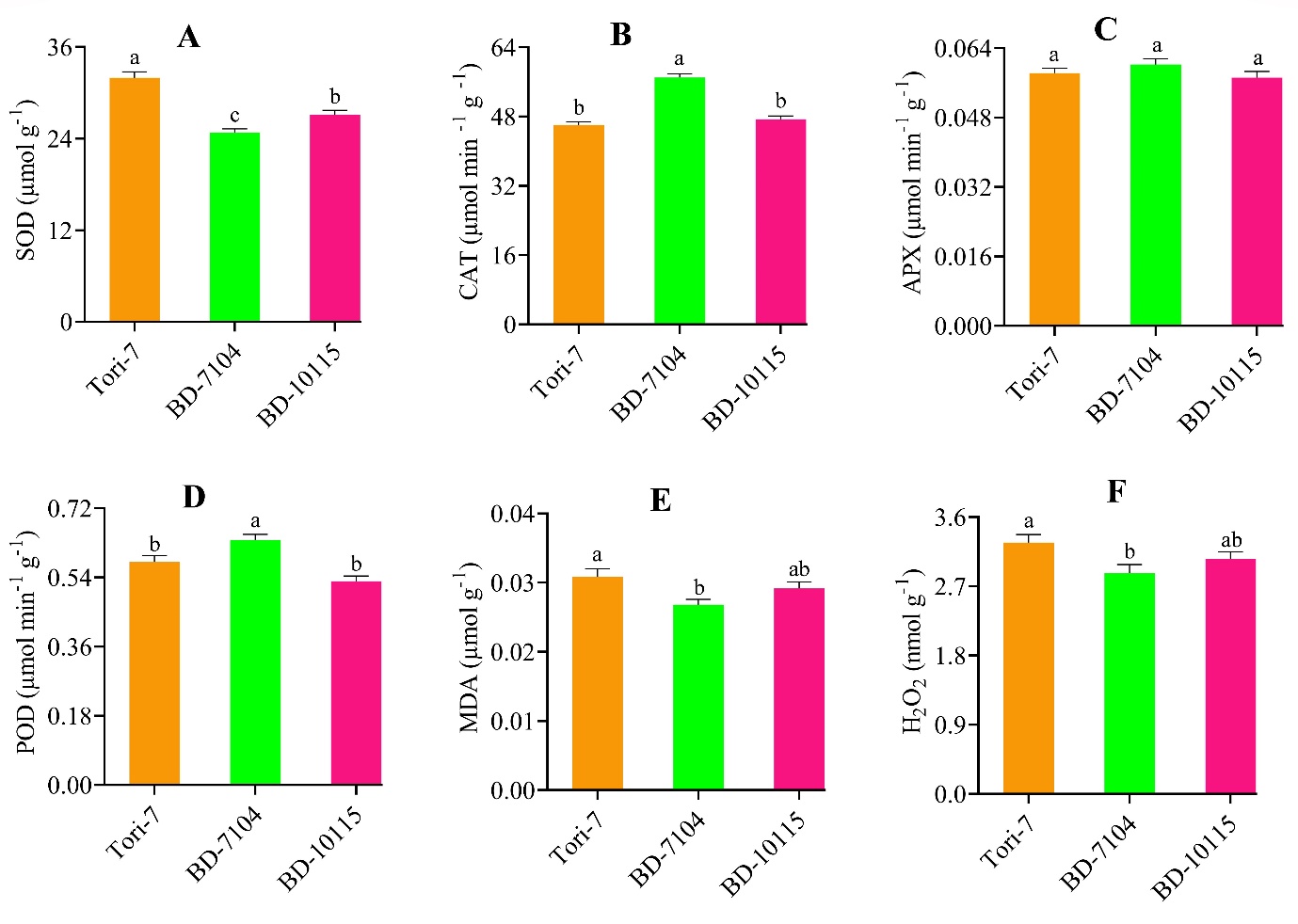


**SI Fig. 2.** SOD = Superoxide dismutase, CAT = Catalase, APX = Ascorbate peroxidase, POD = Peroxidase, MDA = Malondialdehyde, and H_2_O_2_ content of Indian mustard genotypes. The bars are made using mean ± standard error of the mean. The same letter on the treatment bar indicates the statistical similarity among them as per Tukey’s test at p < 0.05.

**SI Table 1**

Growth and yield of Indian mustard genotypes and *T. harzianum* treatment. All the values express mean ± standard error of the mean (n=4). The same letter on the values in a column indicates the statistical similarity among them as per Tukey’s test at p < 0.05.

|  | Plant height  (cm) | No. of branches  plant ^–1^ | No. of siliqua  plant ^–1^ | Seed yield  pot ^–1^ | Stover yield  pot ^–1^ |
| --- | --- | --- | --- | --- | --- |
| Genotype |  |  |  |  |  |
| Tori-7 | 47.17±0.77b | 4.67±0.14b | 154±7b | 5.51±0.16c | 10.74±0.33c |
| BD-7104 | 64.13±0.58a | 5.24±0.19a | 240±7a | 10.33±0.18a | 20.96±0.64a |
| BD-10115 | 62.00±0.89a | 4.86±0.13ab | 233±8a | 8.46±0.16b | 17.12±0.50b |
| *T. harzianum* treatment | |  |  |  |  |
| Td_0_ | 55.04±3.62b | 4.22±0.22b | 179±18b | 7.05±0.71b | 13.48±1.21c |
| TdC_7.5_ | 58.85±2.25ab | 4.67±0.24ab | 206±21a | 8.23±0.75a | 15.99±1.66abc |
| TdC_10.0_ | 59.88±2.44a | 5.11±0.11ab | 220±19a | 8.60±0.78a | 17.03±1.62ab |
| TdC_12.5_ | 60.36±2.44a | 5.44±0.29a | 228±16a | 8.65±0.77a | 18.33±1.99a |
| TdS_110×10_^5^ | 55.98±3.05ab | 4.67±0.24ab | 199±17ab | 7.85±0.69ab | 15.35±1.47bc |
| TdS_110×10_^6^ | 57.57±2.85ab | 5.33±0.24a | 217±16a | 8.21±0.73a | 17.60±1.73ab |
| TdS_110×10_^7^ | 56.69±3.04ab | 5.00±0.17ab | 213±13a | 8.10±0.66a | 16.14±1.48abc |

Td = *Trichoderma*, TdC = *Trichoderma* compost, TdS = *Trichoderma* suspension

NS indicates non-significant at 5% level of significance


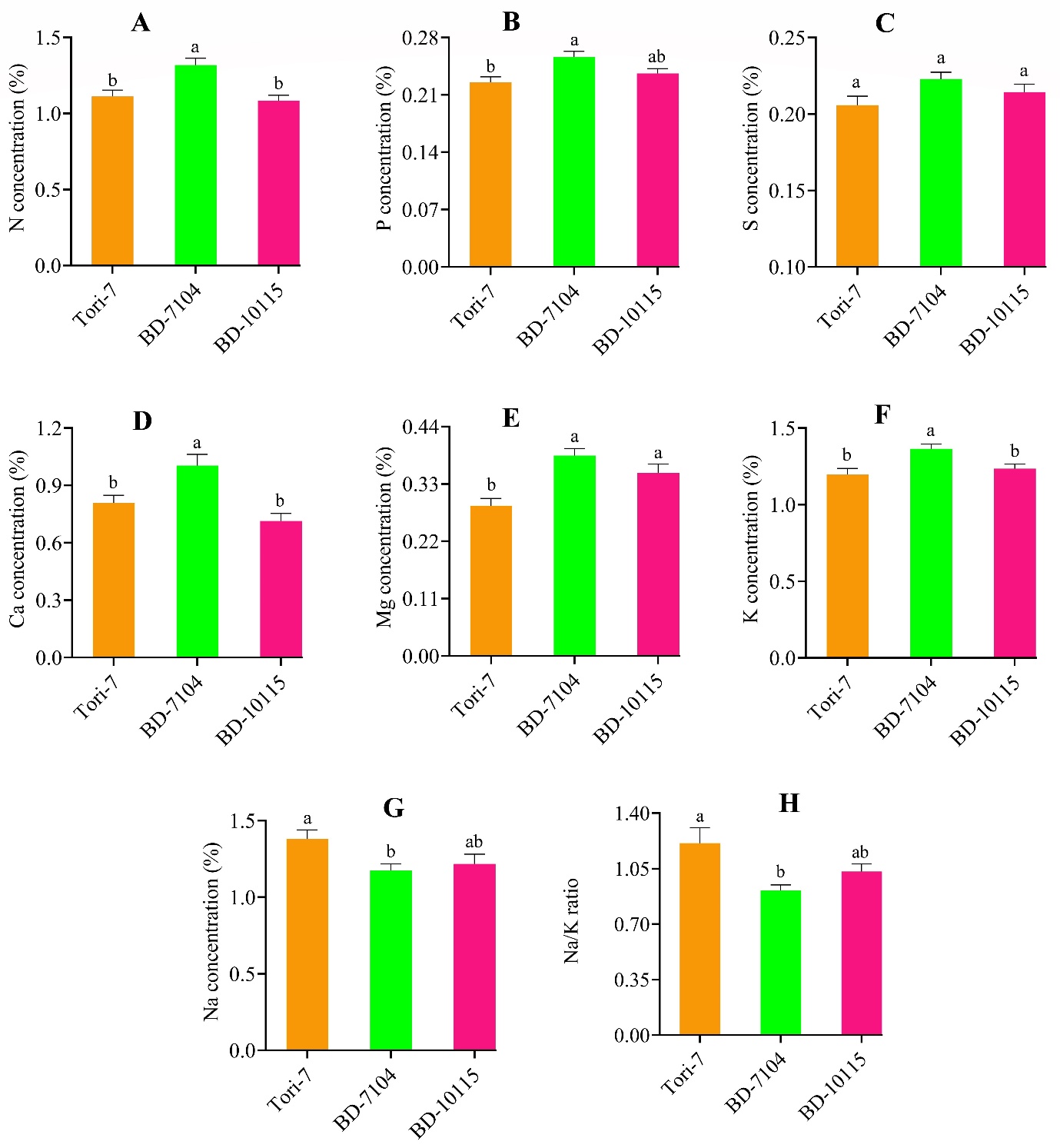


**SI Fig. 3.** Biomass N, P, S, Ca, Mg, K, Na concentration and Na/K ratio of Indian mustard genotypes. The bars are made using mean ± standard error of the mean. The same letter on the treatment bar indicates the statistical similarity among them as per Tukey’s test at p < 0.05.
